# Maternal Attachment Networks and Mother–Infant Bonding Disturbances among Mothers with Postpartum Major Depression

**DOI:** 10.3390/ijerph20126155

**Published:** 2023-06-16

**Authors:** Stéphanie Vanwalleghem, Raphaële Miljkovitch, Aino Sirparanta, Camille Toléon, Stéphanie Leclercq, Anne-Sophie Deborde

**Affiliations:** 1 Unité de Recherche CLIPSYD, Paris Nanterre University, 200 Avenue de la République, 92000 Nanterre, France; 2 Laboratoire Paragraphe, Paris 8 University, 2 Rue de la Liberté, 93200 Saint-Denis, France; raphaele.miljkovitch@iedparis8.net (R.M.); aino.sirparanta02@univ-paris8.fr (A.S.); camille.toleon02@univ-paris8.fr (C.T.); anne-sophie.deborde@univ-paris8.fr (A.-S.D.); 3 Centre Hospitalier la Chartreuse, Unité Père-Mère-Bébé, 1 Boulevard Chanoine Kir, 21000 Dijon, France; stephanie.leclercq@chlcdijon.fr

**Keywords:** attachment, partner, postpartum depression, mother–infant bonding

## Abstract

The literature suggests that maternal insecure attachment is a risk factor for postpartum depression which, in turn, affects motherinfant bonding. However, recent research in attachment suggests that the investigation of attachment networks provides further insight in the understanding of psychological outcomes. This study aims to test a model according to which mothers’ attachment towards each of their parents contributes to explain attachment towards their romantic partners, which itself is associated with maternal postpartum depression and, in turn, with motherinfant bonding. The Attachment Multiple Model Interview, the Edinburgh Postnatal Depression Scale, and the Postpartum Bonding Questionnaire were administered to 90 mothers of infants under 6 months of age (32 with postpartum major depression). Results showed that attachment towards the partner (1) is best explained by attachment to the father and (2) mediates the link between attachment to the father and depression severity. Also, depression severity mediates the link between attachment to the partner and motherinfant bonding. These results highlight the role of attachment models towards the romantic partner and the father in the perinatal period and the relevance of attachment-focused therapeutic programs in treating postpartum maternal depression.

## 1. Introduction

Perinatal depression is an episode of major depression with a peri-partum onset (i.e., during pregnancy or in the four weeks following delivery [1]). Its prevalence is estimated at 11.9% during the whole perinatal period [2] and 17.7% during the postpartum period [3]. One of the well-known risk factors of perinatal depression is maternal insecure attachment [4,5,6,7,8,9,10,11,12,13], and one of its main outcomes is poor maternal bonding [14,15,16,17]. However, studies are lacking when it comes to the specific attachment relationships involved in feelings of maternal insecurity. Recent research on attachment suggests that the investigation of attachment networks provides further insight into the understanding of psychological outcomes. This study aims to examine whether mothers’ attachment towards each of their parents contributes to explain their attachment towards their romantic partner, which itself is linked with postpartum depression. It also aims to examine whether attachment to the romantic partner contributes to explain mother–infant bonding disturbances via maternal postpartum depression. Results are expected to provide directions for care programs of postpartum depression and/or bonding disturbances.

### 1.1. Maternal Bonding and Postpartum Depression

Maternal bonding refers to the maternal emotions and thoughts toward one’s infant [18]. It is thought to develop during pregnancy or immediately after birth [18], and to evolve over the first months of the infant’s life [19]. Bonding includes maternal behaviors such as proximity seeking, touching, contact behavior, gazing, baby talk, positive expression, cuddling, smiling, and adaptation to cues expressed by the infant [17]. Maternal depression is associated with mother–infant bonding disturbances [14,15,16,17]. Difficulties in concentrating, anhedonia, and lack of energy caused by depression [1] may limit the mother’s attention to the infant’s needs and interfere with learning to interpret his/her signals, leading to reduced caregiving skills. Recent studies have shown that perinatal depression is associated with low maternal sensitivity [20,21], low structuring behavior [22], a more intrusive/controlling or unresponsive/passive interaction style [21,23], and less warmth [24]. Insecure attachment style [4,5,6,7,8,9,10,11,12,13] and insecure attachment towards the partner [25] have been identified as risk factors for depression and for associated bonding disturbances. Insecure attachment refers to the feeling of not receiving the attention and support one is in need of [26].

### 1.2. Postpartum Depression and Maternal Attachment Models

Insecure attachment towards the partner has been found to be linked to postpartum depression [25]. According to attachment theory [26,27], attachment in adulthood is influenced by the “internal working models” (IWM) of relationships developed early in life. It is assumed that attachment experiences are encoded in long-term memory as mental models of relationships, which guide perception and behavior in new social situations [28]. Studies showing that attachment towards the partner is predicted by attachment with the mother during childhood [29,30] tend to further corroborate this assumption.

More recent research also suggests that attachment in different relationships explains psychological outcomes better than attachment to a single person [31,32,33]. Consequently, investigating the combined or cascade effects of young mothers’ relationship-specific attachment models (to mother, father, and romantic partner) can be worthwhile to better understand postpartum depression. It can be expected that mothers’ attachments to each of their parents significantly explain attachment to their romantic partners, which itself is associated with postpartum depression.

### 1.3. Maternal Attachment Models, Postpartum Depression and Mother–Infant Bonding

**Figure 1:**
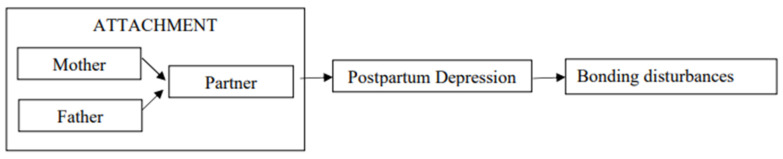
Conceptual mediation model.

Recent studies suggest that maternal insecure attachment not only increases the risk of postpartum depression but may also lead to bonding disturbances. More specifically, researchers have found that maternal postpartum depression partially mediates the link between maternal attachment style and mother–infant bonding [16,34]. These findings suggest that a vicious circle may develop: the less support mothers feel from their attachment relationships, the more depressed they feel and the less able they are to form a high-quality bond with their babies. Thus, a more complex model (see Figure 1) of mother–infant bonding disturbances as being explained by a cascade effect of attachments towards each parent on attachment towards the romantic partner and in turn, on postpartum depression can be hypothesized. Different attachment-focused therapeutic programs have been developed and used to improve mother–infant bonding among mothers with postpartum depression [35,36,37,38,39,40]. Better understanding the factors involved in this disorder and associated bonding disturbances seems important to better specify which interventions are relevant and what the main therapeutic targets should be (infant–mother bonding, mother’s attachment towards each of her parents and/or towards her romantic partner).

### 1.4. The Current Study

Taking into account the links between postpartum depression, maternal attachment, and mother–infant bonding, the objectives of the present study are to test whether (see Figure 1):

1. Mothers’ attachment to each parent is linked to attachment to their romantic partner;
2. Attachment to the partner is linked to depression and mediates the link between attachment to parents and maternal depression;
3. Attachment to the partner contributes to explain mother–infant bonding via depression;
4. Insecure attachment towards each parent contributes to mother–infant bonding disturbances via attachment to the partner and, in turn, via depression.

## 2. Method

### 2.1. Participants and Procedure

Ninety mothers of infants under 6 months of age participated in the study, 32 of whom had a major postpartum depression disorder. Participants with a major postpartum depression disorder were recruited from a psychiatric father–mother–infant ward. Additional participants were recruited via a network of midwives and through social networks in order to constitute a sample with various levels of depressive symptoms. Mothers were recruited between 2020 and 2022, during and after the COVID-19 pandemic. Age of participants ranged from 23 to 42 years (M (Mean) = 31.8; SD (standard deviation) = 4.22). Participants’ socio-economic scores ranged from 12.5 to 66 (M = 45.5; SD = 12.2): 4 participants were in the lowest socio-economic category, 41 were in the mid-range category, and 32 were in the highest category (information was missing for 13 participants). Forty-nine women were multiparous and forty-one were primiparous. Thirty had experienced at least one obstetric labor complication: fifteen had experienced a cesarean section, five reported bleeding, and fourteen the use of instruments (suction cup or forceps). None of the infants had been placed in a neonatal intensive care unit and all of them were born after 37 weeks of pregnancy.

Informed consent was obtained from all the participants. They were free to withdraw from the study at any time without any consequences for their care. Participants filled in the Edinburgh Postnatal Depression Scale and the Postpartum Bonding Questionnaire, passed the Attachment Multiple Model Interview (AMMI; presented below), and answered a few questions on their socio-economic status. The AMMI was administered individually by a trained interviewer. After the interview, participants were offered the possibility to debrief: they had the opportunity to ask questions if they had any, or express their feelings if they had been emotionally disturbed by the experimental protocol. The study was conducted in accordance with the terms of the institution’s ethics and those of the Helsinki World Medical Association Declaration on Ethical Principles for Medical Research involving Human Subjects. The procedure was approved by the Ethics Committee of the psychology department of the university.

### 2.2. Measures

#### 2.2.1. Edinburgh Postnatal Depression Scale 

The Edinburgh Postnatal Depression Scale, EDPS [41], is the most commonly used depression screening tool in perinatal care [42]. It is a 10-item self-report questionnaire that is scored on a Likert scale from 0 to 3, with some items being reverse scored. It provides a continuous depression severity score varying from 0 to 30. Cut-off values of 13 or more are often used to identify women likely to have a depression [43], although a cut-off of 11 seems to maximize sensitivity and specificity [44]. In this study the EDPS score was used as a continuous measure. A literature review of 47 validation studies of the EDPS demonstrated its validity in screening perinatal depression, good sensitivity (ranging from 0.65 to 1.00 depending on the study) and specificity (ranging from 0.71 to 0.97) [45].

#### 2.2.2. Attachment Multiple Model Interview

Attachment representations of specific relationships were assessed with a semi-structured interview, the Attachment Multiple Model Interview (AMMI) [29]. Attachment representations for each relationship (mother, father, partner) were coded on four attachment dimensions: security, deactivation, hyperactivation, and disorganization of the attachment system. Scores of security, deactivation, and hyperactivation vary from 0 to 8, whereas scores of disorganization vary from 0 to 16. The validation study demonstrated that the AMMI significantly discriminates internal working models specific to different relationships [29]. Its validity has also been established with longitudinal data gathered from age 4 to 23, suggesting that AMMI scores at age 23 reflect corresponding cumulated lifetime scores for security, deactivation, and hyperactivation [29]. Also, theoretically consistent links between disorganization and unresolved trauma, maltreatment severity, suicidal risk, and child abuse [29,31,46] further confirm its validity.

#### 2.2.3. Postpartum Bonding Questionnaire (PBQ)

The quality of mother/infant bonding was assessed with the Postpartum Bonding Questionnaire (PBQ [15,47]). The PBQ is a 25-item self-report questionnaire designed to tap disturbances in the mother–infant relationship. Items are scored on a six-point scale from 0 (always) to 5 (never), with some items being reversed. In this study, we focused on the general factor assessing the quality of the mother–infant relationship. The validation study of Brockington et al. [15] demonstrated that this general factor had satisfactory sensitivity (0.82) in identifying a variety of problems in the mother–infant relationship, including sensitivity in identifying the mothers who were likely to have dangerous behaviors towards their infants (contrary to other scales of the PBQ). It is based on 12 questions with scores ranging from 0 to 60. The higher the score, the more severe the disorder.

#### 2.2.4. Socio-Economic Status

Socio-economic status (SES) was measured using Barratt’s simplified measure of social status (BSMSS [48]), based on the profession and the level of education of mothers and their partners and on their marital status (e.g., married). BSMSS scores ranged from 8 to 66 (a higher socio-economic status).

### 2.3. Data Analysis

To determine whether SES (BSMSS) had to be controlled for in the main analyses, correlation analyses were conducted to examine the links between SES and attachment dimensions, mother–infant bonding, and depression. To test whether severity of the depression and infant-mother bonding disturbances increased with insecurity within each attachment relationship, we examined correlations between attachment scores for each relationship (mother, father, partner) and, respectively, depression scores and bonding disturbance scores. Finally, Partial Least Squares-Path Modeling analyses (PLS-PM) were conducted to test the mediation model, according to which depression severity mediates the link between attachment and mother–infant bonding.

## 3. Results

### 3.1. Preliminary Analyses

Concerning socio-economic status, the BSMSS scores were not correlated with any of the attachment scores, nor with bonding or depression scores (all *r_s_* < 0.15, all *p_s_* > 0.10). Consequently, socio-economic status was not controlled for in the main analyses.

### 3.2. Main Analyses

#### 3.2.1. Attachment Insecurity in Each Relationship and Postpartum Depression

Correlations between each attachment score (security, deactivation, hyperactivation, disorganization) for each relationship (mother, father, partner) and depression are presented in Table 1. Expected correlations were found for each attachment dimension in each relationship, except for security with the father, for which no significant link was found. More specifically, depression was otherwise negatively associated with security and positively with deactivation, hyperactivation, and disorganization (see Table 1).

Correlations between each attachment score (security, deactivation, hyperactivation, disorganization) for each relationship (mother, father, partner) and bonding disturbances score are also presented in Table 1. Positive correlations were found between bonding disturbances and hyperactivation toward the father and deactivation toward the partner (see Table 1).

#### 3.2.2. Mediation Model between Attachment to Each Parent and Mother–Infant Bonding via Attachment to the Partner and in Turn via Depression Severity

Partial Least Squares Path Modeling (PLS-PM [49]) was used to test our conceptual mediation model. The analyses were conducted in R version 4.1.2 (The R Foundation for Statistical Computing, Vienna, Austria) [50] using the PLS-PM package [51]. PLS-PM is a variance-based structural equation modeling technique that does not rely on normality assumptions and can be used with small sample sizes [52]. In the PLS-PM approach, two models were tested: (1) the outer (measurement) model describes the relationships with the manifest variables (MVs) and their latent variables (LVs), and (2) the inner (structural) model describes the relationships between the latent variables. To assess the significance of coefficients, bootstrapping technique was used.

The initial conceptual model was composed of 14 manifest variables (MVs) loaded on 5 latent variables (LVs) (see Table 2). Regarding the manifest variables loaded onto the attachment LVs, attachment security scores were inverted to load in the same direction as the deactivation, hyperactivation and disorganization scores: they were converted into insecurity scores. As results indicated that the outer model was acceptable regarding the unidimensionality of all the LVs (all Dillon-Goldstein’s rho > 0.90) and cross-loadings (see Table 3), the final outer model was identical to the initial conceptual model. It comprised 14 MVs loaded on 5 LVs, corresponding to the following factors: depression, bonding disturbances, attachment to mother, attachment to father, attachment to partner.

**Figure 2:**
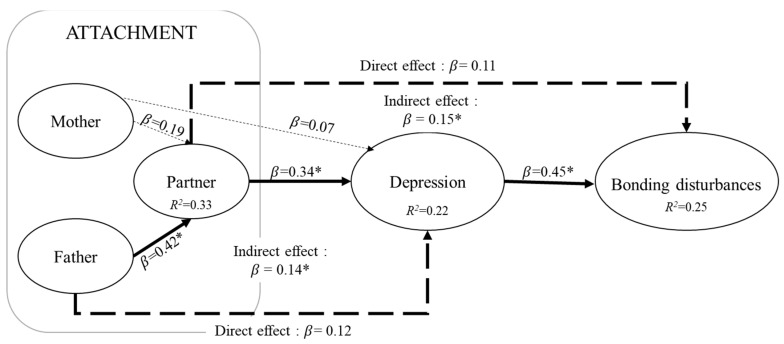
PLS PM graph predicting mother–infant bonding disturbances. Note: * *p* < 0.05.

The inner model (see Table 3) was then built to examine the links between the attachments towards the mother, the father, and the partner, depression, and mother–infant bonding disturbances. Results indicated that Goodness of Fit of the model was 0.44. Direct and indirect bootstrapped path coefficients (95% confidence interval) are presented in Table 4. The model explained 25% of the variance of mother–infant bonding disturbances. Figure 2 shows the path coefficients (*β*) between the LVs for the inner model. Significant direct links were found between attachment to father and attachment to partner (*β* = 0.42), between attachment to partner and depression (*β* = 0.34), and between depression and bonding disturbances (*β* = 0.45). Conversely, there were no direct links between (1) attachment to mother and attachment to partner (*β* = 0.20), nor (2) between bonding disturbances and, respectively, attachment to mother (*β* = −0.24), attachment to father (*β* = 0.13), and attachment to partner (*β* = 0.11). In this model, no direct links were found between depression and (1) attachment to mother (*β* = 0.07) or attachment to father (*β* = 0.12).

However, indirect paths (Table 4) suggest that the indirect effect of attachment to father on depression via attachment to partner (*β* = 0.14) was significant, as well as the indirect effect of attachment to the partner on mother–infant bonding disturbances via depression *(β* = 0.15). However, the mediation linking attachment to mother to bonding disturbances via attachment to partner and depression was not (*β* = 0.08), nor was the mediation linking the attachment to father to bonding disturbances via attachment to partner and depression (*β* = 0.16).

## 4. Discussion

The objective of this study was to test a model, according to which (1) maternal attachment towards the mother and the father contribute to explain attachment to the romantic partner, (2) insecure attachment with the romantic partner is linked to depression severity and, in turn, to impaired mother–infant bonding, and (3) attachment to the mother and attachment to the father are indirectly linked to mother–infant bonding disturbances via attachment to the romantic partner and, in turn, depression severity.

The results first showed that attachment to the romantic partner was linked to attachment to the father, but not to attachment to the mother. These results contrast with the traditional idea of a hierarchical model of attachment formulated by Van IJzendoorn et al. [53] in which the mother is considered more determining than the father (see also Bowlby [54], Main [55], Bretherton and Munholland [56]). They are also different from the findings of Grossmann et al. [30] and of Miljkovitch et al. [29] who found attachment to the partner to be associated with attachment to the mother, but not to the father [29]. In these two studies, participants were young adults, most of whom were not yet parents and probably still living with their parents. Conversely, in the present study, participants were all mothers and were on average 31 years old. The discrepancy between our results and those of the two other studies could be explained by special maternal needs during the perinatal period. During this period, mothers need their romantic partners both as a source of support for themselves and as a father for their newborn child. According to attachment theory [26], attachment to parents influence relationships with others throughout life, namely, couple relationships in adulthood [57]. The present findings bring further insight into the processes at play as they suggest that depending on the context and the circumstances (e.g., young adulthood versus motherhood), specific attachment IWMs could be activated over others.

Concerning the links between attachment and depression, the results showed that insecure maternal attachment models towards the mother, the father, and the romantic partner were all related to the severity of postpartum depression. This is consistent with numerous studies suggesting that insecure attachment is a risk factor for postpartum depression [4,5,6,7,8,9,10,11,12,13]. However, when all the attachment relationships were considered together, only attachment with the romantic partner was directly linked to maternal depression severity. Attachment to the father was indirectly linked to depression via attachment to the romantic partner whereas attachment to the mother was not linked to depression. This result highlights an activation of representations of specific relationships, that is, with the partner and accordingly, with the father. This may be due to mothers’ specific needs during the postpartum period, where the partner plays an important role as a co-parent and source of support, and where questions regarding fatherhood are especially likely to arise.

As expected, maternal depression mediated the link between insecure maternal attachment to the partner and bonding disturbances with the infant: the poorer the quality of attachment to the partner, the higher the risk of postpartum depression and, in turn, of mother–infant bonding disturbances. This result is in line with other studies documenting links between maternal current adult attachment style and mother–infant bonding [58] and with those showing that maternal depression partially mediates the link between attachment and bonding [16,34]. The investigation of specific attachment models of the mother, namely, with her own mother, father, and romantic partner, brings new insight on the way representations of specific relationships seem more or less activated in the postpartum period. They suggest that attachment models are not equally related to bonding and that attachment to the romantic partner has a pivotal role for mothers in the perinatal period. Thus, attachment to the partner may be a relevant therapeutic target in treating postpartum depression. This result is also consonant with studies demonstrating that parental attachment insecurity is associated with reduced involvement [58], less sensitive and responsive parenting behaviors [59,60,61], and less feelings of closeness to the child [62,63]; and with studies showing that support from the romantic partner and the quality of the relationship with him are protective factors for postpartum depression [64,65]. The results of the present study add to previous studies by suggesting that feeling secure with one’s partner may be critical for the emotional well-being of young mothers, as well as for bonding with their infant. They suggest that therapeutic work should not solely be focused on mother–infant bonding, but also on the relationship between the mother and her partner. Moreover, the hypothesis of a possible replication of the model with the father in the relationship with the partner (suggested by the link found between the two) could also be explored by therapists.

Limitations regarding this study should be addressed. First, the correlation sizes were small to moderate, as were the explained variances in the mediation model, suggesting that other factors contribute to the development of postpartum depression and bonding. Secondly, although the PLS-PM analysis tested a model with specified directional links, the cross-sectional design of the study calls for caution as to actual causal effects. For example, depression may cause more negative perceptions of attachment relationships due to cognitive distortions of negativity associated with the disorder. Interestingly, however, the study shows that some relationships are more closely related to postpartum depression than others. Thirdly, mothers were recruited during and after the COVID-19 pandemic. The pandemic has been shown to have a detrimental effect on the quality of life of pregnant women [66] and to be related to psychological outcomes for postpartum women [67,68]. Namely, the prevalence of postpartum depression was higher during the pandemic [67] and mothers who could not be accompanied by their partner during and after childbirth because of COVID restrictions on maternity visits were more at risk of anxiety and postpartum traumatic stress symptoms [68]. The COVID-19 pandemic may thus be a confounding factor in our study and the results may have been affected by the increased distress linked to the COVID-19 context.

## 5. Conclusions

The results of this study highlight the central role of attachment to the partner in the context of postpartum depression and suggest that attachment to the father may contribute to how the relationship with the partner is perceived or vice versa. Beyond the fact that representations of the father significantly account for representations of the partner, the latter are involved in the severity of postpartum depression, which, in turn, is associated with an increased risk of disturbances in mother–infant bonding. This underlines the pertinence of orienting therapy on the relationship with the romantic partner and on how it may be tinted by the attachment model of the relationship with the father. Hence, therapeutic programs which focus on the attachment with the romantic partner, such as emotionally focused couple therapy [35], which aims to strengthen the relationship between the mother and her partner, seem particularly relevant for the treatment of postpartum depression and can be an interesting complement to programs focused on the mother–infant relationship.

## Figures and Tables

**Table 1:** Correlations between depression and attachment scores for each relationship.

		Depression	Bonding Disturbances
Attachment	Dimensions		
Mother	Security	−0.24 *	0.01
	Deactivation	0.24 *	0.04
	Hyperactivation	0.29 **	0.07
	Disorganization	0.30 **	0.09
Father	Security	−0.19	0.01
	Deactivation	0.39 ***	0.17
	Hyperactivation	0.30 **	0.22 *
	Disorganization	0.32 **	0.17
Romantic partner	Security	−0.36 ***	−0.18
	Deactivation	0.29 **	0.26 *
	Hyperactivation	0.43 ***	0.19
	Disorganization	0.36 ***	0.19

Note. * *p* < 0.05. ** *p* < 0.01. *** *p* < 0.001.

**Table 2:** Descriptive statistics for latent variables (LVs) and manifest variables (MVs).

Latent Variables (LVs)	Manifest Variables (MVs)	Mean (SD)
Depression	Depression	9.69 (7.20)
Bonding disturbances	Bonding disturbances	13.4 (15.3)
Attachment Mother	Security mother	4.67 (2.16)
	Deactivation mother	3.66 (2.31)
	Hyperactivation mother	2.86 (2.09)
	Disorganization mother	4.74 (4.13)
Attachment Father	Security father	4.53 (2.01)
	Deactivation father	3.66 (2.38)
	Hyperactivation father	2.20 (1.83)
	Disorganization father	4.13 (3.76)
Attachment Partner	Security partner	5.80 (1.43)
	Deactivation partner	2.28 (1.70)
	Hyperactivation partner	2.96 (1.81)
	Disorganization partner	3.75 (3.18)

**Table 3:** Unidimensionality test for latent variables (LVs) and manifest variables (MVs).

Latent Variables (LVs)	Manifest Variables (MVs)	Unidimensionality
Depression	Depression	1
Bonding disturbances	Bonding disturbances	1
Attachment Mother	Insecurity mother	0.92
	Deactivation mother	
	Hyperactivation mother	
	Disorganization mother	
Attachment Father	Insecurity father	0.92
	Deactivation father	
	Hyperactivation father	
	Disorganization father	
Attachment Partner	Insecurity partner	0.90
	Deactivation partner	
	Hyperactivation partner	
	Disorganization partner	

**Table 4:** Direct and indirect bootstrapped path coefficients.

Latent Variables (LVs)	*β* Weight			95% Bootstrap CI
Attachment-Mother → Attachment-Partner	0.19			[−0.04, 0.40]
Attachment-Father → Attachment-Partner	0.42			[0.21, 0.64] *
Attachment-Mother → Depression	0.07			[−0.22, 0.36]
Attachment-Mother → Bonding	−0.24			[−0.52, 0.07]
Attachment-Father → Depression	0.12			[−0.13, 0.41]
Attachment-Father → Bonding	0.13			[−0.21, 0.42]
Attachment-Partner → Depression	0.34			[0.10, 0.56] *
Attachment-Partner → Bonding	0.11			[−0.07, 0.32]
Depression → Bonding	0.45			[0.20, 0.70] *
Direct and indirect paths	Direct	Indirect	Total	
Attachment-Mother → Attachment-Partner → Depression	0.07	0.07	0.14	[−0.17, 0.44]
Attachment-Father → Attachment-Partner → Depression	0.12	0.14	0.26	[0.01, 0.55] *
Attachment-Partner → Depression → Bonding	0.11	0.15	0.26	[0.06, 0.51] *
Attachment-Mother → Attachment-Partner → Depression → Bonding	−0.24	0.08	−0.16	[−0.49, 0.27]
Attachment-Father → Attachment-Partner → Depression → Bonding	0.13	0.16	0.29	[−0.06, 0.60]

Note: CI = Confidence interval; * *p* < 0.05.

## Data Availability

The data that support the findings of this study are available from the corresponding author, S.V., upon reasonable request.

## References

[1] American Psychiatric Association (2013). Diagnostic and Statistical Manual of Mental Disorders.

[2] Woody C.A., Ferrari A.J., Siskind D.J., Whiteford H.A., Harris M.G. (2017). A Systematic Review and Meta-Regression of the Prevalence and Incidence of Perinatal Depression. J. Affect. Disord..

[3] Hahn-Holbrook J., Cornwell-Hinrichs T., Anaya I. (2018). Economic and Health Predictors of National Postpartum Depression Prevalence: A Systematic Review, Meta-Analysis, and Meta-Regression of 291 Studies from 56 Countries. Front. Psychiatry.

[4] Axfors C., Sylvén S., Ramklint M., Skalkidou A. (2017). Adult Attachment’s Unique Contribution in the Prediction of Postpartum Depressive Symptoms, beyond Personality Traits. J. Affect. Disord..

[5] Bifulco A., Figueiredo B., Guedeney N., Gorman L.L., Hayes S., Muzik M., Glatigny-Dallay E., Valoriani V., Kammerer M.H., Henshaw C.A. (2004). Maternal Attachment Style and Depression Associated with Childbirth: Preliminary Results from a European and US Cross-Cultural Study. Br. J. Psychiatry.

[6] Feeney J., Alexander R., Noller P., Hohaus L. (2003). Attachment Insecurity, Depression, and the Transition to Parenthood. Pers. Relatsh..

[7] Hairston I.S., Handelzalts J.E., Assis C., Kovo M. (2018). Postpartum bonding difficulties and adult attachment styles: The mediating role of postpartum depression and childbirth-related ptsd: Postpartum Bonding and Adult Attachment Styles. Infant Ment. Health J..

[8] McMahon C., Barnett B., Kowalenko N., Tennant C. (2005). Psychological Factors Associated with Persistent Postnatal Depression: Past and Current Relationships, Defence Styles and the Mediating Role of Insecure Attachment Style. J. Affect. Disord..

[9] Sabuncuoğlu O., Berkem M. (2006). Relationship between attachment style and depressive symptoms in postpartum women: Findings from Turkey. Turk Psikiyatr. Derg. Turk. J. Psychiatry.

[10] Simpson J.A., Rholes W.S., Campbell L., Tran S., Wilson C.L. (2003). Adult Attachment, the Transition to Parenthood, and Depressive Symptoms. J. Pers. Soc. Psychol..

[11] McMahon C.A., Barnett B., Kowalenko N.M., Tennant C.C. (2006). Maternal Attachment State of Mind Moderates the Impact of Postnatal Depression on Infant Attachment. J. Child Psychol. Psychiatry.

[12] McMahon C., Trapolini T., Barnett B. (2008). Maternal State of Mind Regarding Attachment Predicts Persistence of Postnatal Depression in the Preschool Years. J. Affect. Disord..

[13] Smith-Nielsen J., Steele H., Mehlhase H., Cordes K., Steele M., Harder S., Væver M.S. (2015). Links among High EPDS Scores, State of Mind Regarding Attachment, and Symptoms of Personality Disorder. J. Pers. Disord..

[14] Kasamatsu H., Tsuchida A., Matsumura K., Shimao M., Hamazaki K., Inadera H., The Japan Environment and Children’s Study Group (2020). Understanding the Relationship between Postpartum Depression One Month and Six Months after Delivery and Mother-Infant Bonding Failure One-Year after Birth: Results from the Japan Environment and Children’s Study (JECS). Psychol. Med..

[15] Brockington I.F., Fraser C., Wilson D. (2006). The Postpartum Bonding Questionnaire: A Validation. Arch. Womens Ment. Health.

[16] Nonnenmacher N., Noe D., Ehrenthal J.C., Reck C. (2016). Postpartum Bonding: The Impact of Maternal Depression and Adult Attachment Style. Arch. Womens Ment. Health.

[17] Reck C., Klier C.M., Pabst K., Stehle E., Steffenelli U., Struben K., Backenstrass M. (2006). The German Version of the Postpartum Bonding Instrument: Psychometric Properties and Association with Postpartum Depression. Arch. Womens Ment. Health.

[18] Kinsey B.C., Hupcey J.E. (2013). State of the Science of Maternal–Infant Bonding: A Principle-Based Concept Analysis. Midwifery.

[19] Muzik M., Bocknek E.L., Broderick A., Richardson P., Rosenblum K.L., Thelen K., Seng J.S. (2013). Mother–Infant Bonding Impairment across the First 6 Months Postpartum: The Primacy of Psychopathology in Women with Childhood Abuse and Neglect Histories. Arch. Womens Ment. Health.

[20] Musser E.D., Ablow J.C., Measelle J.R. (2012). Predicting Maternal Sensitivity: The Roles of Postnatal Depressive Symptoms and Parasympathetic Dysregulation. Infant Ment. Health J..

[21] Binda V., Figueroa-Leigh F., Olhaberry M. (2019). Antenatal and Postnatal Depressive Symptoms: Association with Quality of Mother–Infant Interaction. Infant Behav. Dev..

[22] Hakanen H., Flykt M., Sinervä E., Nolvi S., Kataja E.-L., Pelto J., Karlsson H., Karlsson L., Korja R. (2019). How Maternal Pre- and Postnatal Symptoms of Depression and Anxiety Affect Early Mother-Infant Interaction?. J. Affect. Disord..

[23] Flykt M., Kanninen K., Sinkkonen J., Punamäki R.-L. (2010). Maternal Depression and Dyadic Interaction: The Role of Maternal Attachment Style. Infant Child Dev..

[24] Humphreys K.L., King L.S., Choi P., Gotlib I.H. (2018). Maternal Depressive Symptoms, Self-Focus, and Caregiving Behavior. J. Affect. Disord..

[25] Iles J., Slade P., Spiby H. (2011). Posttraumatic Stress Symptoms and Postpartum Depression in Couples after Childbirth: The Role of Partner Support and Attachment. J. Anxiety Disord..

[26] Bowlby J. (1973). Attachment and Loss, Vol. 2: Separation.

[27] Bretherton I., Munholland K.A. (2016). The Internal Working Model Construct in Light of Contemporary Neuroimaging Research. Handcook of Attachment.

[28] Booth-LaForce C., Groh A.M., Burchinal M.R., Roisman G.I., Owen M.T., Cox M.J.V. (2014). Caregiving and contextual sources of continuity and change in attachment security from infancy to late adolescence: Caregiving and contextual sources of continuity and change. Monogr. Soc. Res. Child Dev..

[29] Miljkovitch R., Moss E., Bernier A., Pascuzzo K., Sander E. (2015). Refining the Assessment of Internal Working Models: The Attachment Multiple Model Interview. Attach. Hum. Dev..

[30] Grossmann K., Grossmann K.E., Kindler H., Waters E. (2005). Early Care and the Roots of Attachment and Partnership Representations. Attachment from Infancy to Adulthood: The Major Longitudinal Studies.

[31] Touati C.D., Miljkovitch R., Sirparanta A., Deborde A.-S. (2022). The Role of Attachment to the Foster Parent with Regard to Suicidal Risk among Adult Survivors of Childhood Maltreatment. Child Abus. Negl..

[32] Miljkovitch R., Deborde A.-S., Bernier A., Corcos M., Speranza M., Pham-Scottez A. (2018). Borderline Personality Disorder in Adolescence as a Generalization of Disorganized Attachment. Front. Psychol..

[33] Dagan O., Sagi-Schwartz A. (2021). Early Attachment Networks to Multiple Caregivers: History, Assessment Models, and Future Research Recommendations. New Dir. Child Adolesc. Dev..

[34] Handelzalts J.E., Levy S., Molmen-Lichter M., Ayers S., Krissi H., Wiznitzer A., Peled Y. (2021). The Association of Attachment Style, Postpartum PTSD and Depression with Bonding—A Longitudinal Path Analysis Model, from Childbirth to Six Months. J. Affect. Disord..

[35] Johnson S.M., Greenberg L.S. (1985). Differential Effects of Experiential and Problem-Solving Interventions in Resolving Marital Conflict. J. Consult. Clin. Psychol..

[36] Marvin R., Cooper G., Hoffman K., Powell B. (2002). The Circle of Security Project: Attachment-Based Intervention with Caregiver-Pre-School Child Dyads. Attach. Hum. Dev..

[37] Dolhanty J., Greenberg L.S. (2009). Emotion-Focused Family Therapy in a case of anorexia nervosa. Clin. Psychol. Psychother..

[38] Slade A., Holland M.L., Ordway M.R., Carlson E.A., Jeon S., Close N., Mayes L.C., Sadler L.S. (2020). Minding the Baby^®^: Enhancing Parental Reflective Functioning and Infant Attachment in an Attachment-Based, Interdisciplinary Home Visiting Program. Dev. Psychopathol..

[39] Liddle H.A., Dakof G.A., Parker K., Diamond G.S., Barrett K., Tejeda M. (2001). Multidimensional Family Therapy for Adolescent Drug Abuse: Results of a Randomized Clinical Trial. Am. J. Drug Alcohol Abus..

[40] Eyberg S.M., Robinson E.A. (1982). Parent–Child Interaction Training: Effects on Family Functioning. J. Clin. Child Psychol..

[41] Cox J.L., Holden J.M., Sagovsky R. (1987). Detection of Postnatal Depression. Development of the 10-Item Edinburgh Postnatal Depression Scale. Br. J. Psychiatry.

[42] Hewitt C.E., Gilbody S.M., Mann R., Brealey S. (2010). Instruments to Identify Post-Natal Depression: Which Methods Have Been the Most Extensively Validated, in What Setting and in Which Language?. Int. J. Psychiatry Clin. Pract..

[43] Hewitt C., Gilbody S., Brealey S., Paulden M., Palmer S., Mann R., Green J., Morrell J., Barkham M., Light K. (2009). Methods to Identify Postnatal Depression in Primary Care: An Integrated Evidence Synthesis and Value of Information Analysis. Health Technol. Assess..

[44] Levis B., Negeri Z., Sun Y., Benedetti A., Thombs B.D., Skalkidou A. (2020). Accuracy of the Edinburgh Postnatal Depression Scale (EPDS) for Screening to Detect Major Depression among Pregnant and Postpartum Women: Systematic Review and Meta-Analysis of Individual Participant Data. BMJ.

[45] Jardri R. (2004). Le dépistage de la dépression postnatale: Revue qualitative des études de validation de l’Edinburgh Postnatal Dépression Scale. Devenir.

[46] Miljkovitch R., Danner-Touati C., Gery I., Bernier A., Sirparanta A., Deborde A.-S. (2022). The Role of Multiple Attachments in Intergenerational Transmission of Child Sexual Abuse among Male Victims. Child Abus. Negl..

[47] Brockington I.F., Oates J., George S., Turner D., Vostanis P., Sullivan M., Loh C., Murdoch C. (2001). A Screening Questionnaire for Mother-Infant Bonding Disorders. Arch. Womens Ment. Health.

[48] Barratt W. (2006). Barratt Simplified Measure of Social Status (BSMSS).

[49] Vinzi V.E., Trinchera L., Amato S., Vinzi V.E., Chin W.W., Henseler J., Wang H. (2010). PLS Path Modeling: From Foundations to Recent Developments and Open Issues for Model Assessment and Improvement. Handbook of Partial Least Squares: Concepts, Methods and Applications.

[50] R Core Team (2021). R: A Language and Environment for Statistical Computing. R Foundation for Statistical Computing, Vienna, Austria. https://www.R-project.org/.

[51] Sanchez G. (2013). PLS Path Modeling with R.

[52] Chin W.W., Marcolin B.L., Newsted P.R. (2003). A Partial Least Squares Latent Variable Modeling Approach for Measuring Interaction Effects: Results from a Monte Carlo Simulation Study and an Electronic-Mail Emotion/Adoption Study. Inf. Syst. Res..

[53] van Ijzendoorn M.H., Sagi A., Lambermon M.W.E. (1992). The Multiple Caretaker Paradox: Data from Holland and Israel. New Dir. Child Adolesc. Dev..

[54] Bowlby J. (1988). A Secure Base.

[55] Main M. (1999). Epilogue. Attachment Theory: Eighteen Points with Suggestions for Future Studies. Handbook of Attachment: Theory, Research, and Clinical Applications.

[56] Bretherton I., Munholland K.A. (2008). Internal Working Models in Attachment Relationships: Elaborating a Central Construct in Attachment Theory. Handbook of Attachment: Theory, Research, and Clinical Applications.

[57] Hazan C., Shaver P. (1987). Romantic Love Conceptualized as an Attachment Process. J. Pers. Soc. Psychol..

[58] van Bussel J.C.H., Spitz B., Demyttenaere K. (2010). Three Self-Report Questionnaires of the Early Mother-to-Infant Bond: Reliability and Validity of the Dutch Version of the MPAS, PBQ and MIBS. Arch. Womens Ment. Health.

[59] Selcuk E., Günaydin G., Sumer N., Harma M., Salman S., Hazan C., Dogruyol B., Ozturk A. (2010). Self-Reported Romantic Attachment Style Predicts Everyday Maternal Caregiving Behavior at Home. J. Res. Pers..

[60] Edelstein R.S., Alexander K.W., Shaver P.R., Schaaf J.M., Quas J.A., Lovas G.S., Goodman G.S. (2004). Adult Attachment Style and Parental Responsiveness during a Stressful Event. Attach. Hum. Dev..

[61] Mills-Koonce W.R., Appleyard K., Barnett M., Deng M., Putallaz M., Cox M. (2011). Adult Attachment Style and Stress as Risk Factors for Early Maternal Sensitivity and Negativity. Infant Ment. Health J..

[62] Wilson C.L., Rholes W.S., Simpson J.A., Tran S. (2007). Labor, Delivery, and Early Parenthood: An Attachment Theory Perspective. Pers. Soc. Psychol. Bull..

[63] Rholes W.S., Simpson J.A., Blakely B.S. (1995). Adult Attachment Styles and Mothers’ Relationships with Their Young Children. Pers. Relatsh..

[64] Smorti M., Ponti L., Pancetti F. (2019). A Comprehensive Analysis of Post-Partum Depression Risk Factors: The Role of Socio-Demographic, Individual, Relational, and Delivery Characteristics. Front. Public Health.

[65] Collins N.L., Dunkel-Schetter C., Lobel M., Scrimshaw S.C.M. (1993). Social Support in Pregnancy: Psychosocial Correlates of Birth Outcomes and Postpartum Depression. J. Pers. Soc. Psychol..

[66] Biviá-Roig G., La Rosa V.L., Gómez-Tébar M., Serrano-Raya L., Amer-Cuenca J.J., Caruso S., Commodari E., Barrasa-Shaw A., Lisón J.F. (2020). Analysis of the Impact of the Confinement Resulting from COVID-19 on the Lifestyle and Psychological Wellbeing of Spanish Pregnant Women: An Internet-Based Cross-Sectional Survey. Int. J. Environ. Res. Public Health.

[67] Chen Q., Li W., Xiong J., Zheng X. (2022). Prevalence and Risk Factors Associated with Postpartum Depression during the COVID-19 Pandemic: A Literature Review and Meta-Analysis. Int. J. Environ. Res. Public Health.

[68] Oddo-Sommerfeld S., Schermelleh-Engel K., Konopka M., La Rosa V.L., Louwen F., Sommerlad S. (2022). Giving Birth Alone Due to COVID-19-Related Hospital Restrictions Compared to Accompanied Birth: Psychological Distress in Women with Caesarean Section or Vaginal Birth—A Cross-Sectional Study. J. Perinat. Med..

